# Influence of Mental Impressions as a Cause of Bodily Deformity

**Published:** 1865-12

**Authors:** 


### INFLUENCE OF MENTAL IMPRESSIONS AS A CAUSE OF BODILY DEFORMITY.

---

At a recent meeting of the London Obstetrical Society, the *British Medical Journal* says, that Dr. Meadows read the particulars of a case of monstrosity. Having first expressed his conviction in favor of the proposition, that the mind can and does act in this way, he reviewed and combatted the various objections urged against it; the principal one being the absence of any direct connection between the nervous system of the mother and that of the fœtus through the umbilical cord. He, however, endeavored to prove that mind, or the mental force, was not and could not be thus bound down, as it were, by the anatomical limits of the nervous structures; that it must have a power of action, if it has any action at all, throughout the entire organism, and in every part of it, whether it possessed nerves or not: in other words, that its sphere of action was only limited by the configuration of the body. Hence in those tissues where the existence of nervous elements could not be demonstrated, the mental or nerve force might, as it were, pass across the intervening matter between any two parts where nerves did exist, just as electricity traversed space between any two conductors. The author then applied this reasoning to the case of the fœtus *in utero*, and offered an explanation of the mode by which mind thus acts upon matter by supposing a kind of correlation between mental and nerve force analogous to the correlation of other physico-vital forces; the nerve force being here the active agent in those nutritive processes upon the changes of which deformities depend.

Dr. Rasch said that the belief in the influence of mental emotion on the formation of the fœtus was as old as it was general amongst women. The great difficulty was to establish facts, as the mothers always recollected something from their pregnancy *after* they found something abnormal in their children. Dr. R. had seen two cases which had somewhat struck him, and of which he had taken notes. Two boys, from different parts of Germany, were brought to him with scarcely any foreskin, looking exactly like circumcised little Jews, but without any cicatrices. Both women narrated, with great emotion, how they had seen during their pregnancies little Jews so cruelly treated that they could not forget it! They both had been present at the well known Jewish rite. He ought to mention, however, that he had observed the same state in other little boys where no such story was volunteered. and where the mothers *did not know that this state was abnormal.* He considered this influence on the fœtus an open question, which he had made up his mind to help in solving whenever there was an opportunity of doing so.

The President, Dr. Barnes, observed that the instances most strongly bearing upon the question were those in which pregnant women stated explicitly the emotions supposed to influence the form of the fœtus *in utero* which they had undergone. The facts of the cases reported were very remarkable, inasmuch as the event singularly confirmed the statement of the woman made before her child was born. Dr.

Barnes remarked that malformations were common amongst birds as well as quadrupeds; and that in birds it must be concluded that any mental impression must be imparted in the earliest stage of development— *i. e.*, before the ovum was invested with the shell.—*Med. & Surg. Reporter.*

